# Fur in *Magnetospirillum gryphiswaldense* Influences Magnetosomes Formation and Directly Regulates the Genes Involved in Iron and Oxygen Metabolism

**DOI:** 10.1371/journal.pone.0029572

**Published:** 2012-01-04

**Authors:** Lei Qi, Jian Li, WeiJia Zhang, Jiangning Liu, Chengbo Rong, Ying Li, Longfei Wu

**Affiliations:** 1 State Key Laboratory of Agro-biotechnology and College of Biological Sciences, China Agricultural University, Beijing, China; 2 Laboratoire de Chimie Bactérienne, Université de la Méditerranée Aix-Marseille II, Institut de Microbiologie de la Méditerranée, CNRS, Marseille, France; 3 France-China Biomineralization and Nano-structure Laboratory, Beijing, China; Auburn University, United States of America

## Abstract

*Magnetospirillum gryphiswaldense* strain MSR-1 has the unique capability of taking up large amounts of iron and synthesizing magnetosomes (intracellular magnetic particles composed of Fe_3_O_4_). The unusual high iron content of MSR-1 makes it a useful model for studying biological mechanisms of iron uptake and homeostasis. The ferric uptake regulator (Fur) protein plays a key role in maintaining iron homeostasis in many bacteria. We identified and characterized a *fur*-homologous gene (MGR_1314) in MSR-1. MGR_1314 was able to complement a *fur* mutant of *E. coli* in iron-responsive manner *in vivo*. We constructed a *fur* mutant strain of MSR-1. In comparison to wild-type MSR-1, the mutant strain had lower magnetosome formation, and was more sensitive to hydrogen peroxide and streptonigrin, indicating higher intracellular free iron content. Quantitative real-time RT-PCR and chromatin immunoprecipitation analyses indicated that Fur protein directly regulates expression of several key genes involved in iron transport and oxygen metabolism, in addition it also functions in magnetosome formation in *M. gryphiswaldense*.

## Introduction

Iron is an essential microelement for bacteria, being an important cofactor for a wide range of cellular processes, *e.g.*, nitrogen fixation, photosynthesis, H_2_ production and consumption, membrane energetic, oxygen transport and DNA biosynthesis. Despite the fact that iron is the fourth most abundant element in the earth's crust, it is often a limiting nutrient in biological systems because of its poor solubility under physiological conditions [1]. Most microorganisms have consequently evolved special mechanisms to assimilate and utilize iron from the environment. On the other hand, excessive uptake of iron may lead to oxidative damage via the Fenton reaction [2], [3], so precise control of iron homeostasis is necessary. In bacteria, Fur (ferric uptake regulator) is the most common and best characterized transcriptional regulator of genes involved in iron uptake, storage and metabolism. When sufficient iron is present, Fur forms a complex with ferrous ions, and binds to a conserved 19 bp DNA sequence (“Fur box”) which overlaps the promoters and suppresses their transcription. When iron is scarce, Fur dissociates from the promoters, their transcription occurs and genes involved in the iron uptake system are expressed [4], [5].

*Magnetospirillum gryphiswaldense* strain MSR-1 is a freshwater, magnetotactic bacterium belonging to the class alpha-Proteobacteria. MSR-1 has the unique ability to synthesize intracellular magnetic particles (termed magnetosomes) composed of magnetite (Fe_3_O_4_) crystals, and therefore has an extremely high iron requirement, ∼100 times higher than *Escherichia coli*. Clearly, MSR-1 must have precise genetic and physiological mechanisms to balance the high iron levels necessary for magnetosome production, vs. the potential toxic effects of excessive intracellular iron. However, these mechanisms are poorly understood [6], [7].

Here we report identification and analysis of Fur protein in *M. gryphiswaldense*. We cloned a *fur* gene, MGR_1314, and it functionally complements a *fur* mutant strain of *E. coli*. To clarify the role of the Fur protein, termed Fur_MSR_, we constructed a *fur* mutant of *M. gryphiswaldense*, applied quantitative real-time RT-PCR (qRT-PCR) and chromatin immunoprecipitation (ChIP) assays to study Fur-mediated regulation of iron and oxygen metabolism. Fur_MSR_ was shown to directly regulate transcription of *katG* (MGR_4274), *sodB* (MGR_3446) and genes for two Fe^2+^ transport system proteins, *feoAB1* (EF120624.1) and *feoAB2* (MGR_1447-1446) in MSR-1. Furthermore, the *fur* knockout mutant displayed reduced biosynthesis of magnetosomes. Our results suggests that *fur* gene assists in magnetosome formation in MSR-1, that Fur protein directly regulates expression of several genes involved in iron and oxygen metabolism.

## Results

### MGR_1314 of *M. gryphiswaldense* MSR-1 functions as a Fur protein

Examination of the genomic sequence of MSR-1 revealed the presence of four genes (MGR_1305, MGR_1314, MGR_1399, MGR_3480) having products characterized as belonging to the Fur protein family. Previous studies have demonstrated great diversity in metal selectivity and biological function within the Fur family, including sensors of metal (Fur for iron, Zur for zinc, Mur for manganese), of peroxide stress (PerR), and of heme availability (iron response regulator, Irr).

BlastP analysis revealed that, among the above four Fur-like repressors, MGR_1314 has the highest degree of homology to Fur proteins from alpha-Proteobacteria such as *Rhizobium leguminosarum* (83%), *Bradyrhizobium japonicum* (80%), *Sinorhizobium meliloti* (71%), and moderate homology to Fur from gamma-Proteobacteria such as *E. coli* (43%) and *Pseudomonas aeruginosa* (42%). MGR_1314 is 432 bp long, encodes 143 amino acid residues, and has pI 5.88 and deduced molecular weight 16.4 kDa.

Amino acid sequence analysis of MGR_1314 revealed that it is neighbored to a ROS/MUCR transcriptional regulator protein (MGR_1313) and hemolysin (MGR_1315) within the genome. It is not a MAI (Magnetosome Island) gene. It contains all the typical features of Fur proteins: a putative regulatory Fe-sensing site located in the dimerization domain, consisting of H87, D89, E108, and H125; and a Zn-binding site, composed of H33, E81, H90, and E101. MGR_1314 is therefore a promising candidate for Fe-responsive regulator in the Fur family (**Figure S1**).

Comparative analysis of MGR_1314 vs. Fur from *P. aeruginosa* shows that the C-terminal metal binding site is highly conserved [8], whereas there is less similarity for the N-terminal DNA binding site, indicating a difference in DNA binding between the two proteins (**Figure S2**).

To determine whether the MGR_1314 gene of MSR-1 encodes a functional Fur protein, we performed complementation of the *fur*-defective *E. coli* strain H1780 as described by Hantke [9]. H1780 contains a chromosomal *lacZ* gene whose expression is controlled by a promoter directly regulated by Fur, the promoter of catecholate siderophore receptor (fiu, ECDH10B_0873). Because of the *fur* mutation, the *fiu-lacZ* reporter gene can not be repressed, and β-galactosidase is constitutively expressed. H1780 is therefore appropriate for testing the function of a *fur* homologue as an iron-responsive repressor protein.

**Figure 1:**
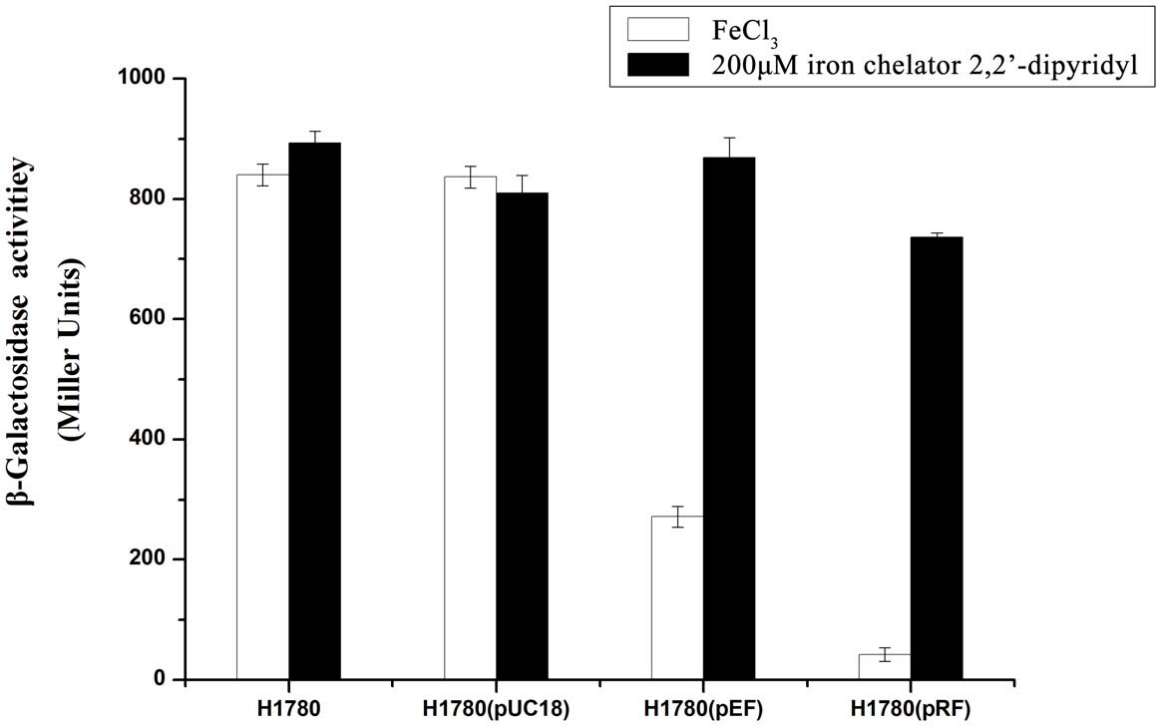
β-galactosidase activity of *E. coli* strain H1780 carrying *fiu*-*lac*Z fusion. Bars represent *fur* mutant (H1780) reporter strain, H1780 containing *E. coli* (pEF) and *M. gryphiswaldense* (pRF) *fur* genes on pUC18 vector, and a vector control. Cells were grown in LB medium with 100 µM FeCl_3_ (black bar), or supplemented with 200 µM DIPy (iron chelator) (white bar). Each assay was performed in three independent experiments, each in triplicate. Values shown are means with S.D., statistically significant (P<0.05) difference for strains grown under low-iron vs. high-iron condition.

In H1780 carrying MGR_1314, expression of fiu-lacZ was significantly (P<0.05) repressed under high-iron condition (**Figure 1**), similarly to Fur from *E. coli*. Based on these findings we concluded that *fur*-like gene MGR_1314 of MSR-1 encodes a functional Fur homologue, which we termed Fur_MSR_.

### *fur* mutant strain (F4) is hypersensitive to H_2_O_2_ and to SNG

**Figure 2:**
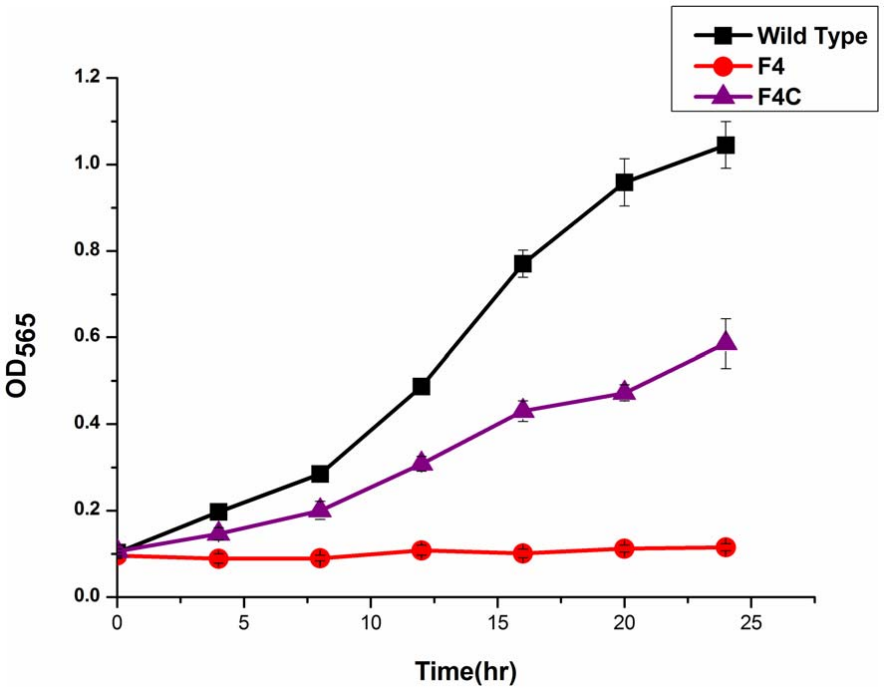
Growth curves measured by OD_565_ of WT, F4, and F4C strains in SLM added with 1 mM H_2_O_2_. Experiments were performed in triplicate, and representative results are shown.

To investigate the function of Fur_MSR_, we constructed a *fur* mutant strain (F4) and its complementation strain (F4C). A common trait in *fur* mutants is increased sensitivity to H_2_O_2_ [10]. We tested the effect of 1 mM H_2_O_2_ on growth of wild-type (WT), F4 and F4C strains (**Figure 2**). H_2_O_2_ has little effect on WT, but inhibited growth of F4. However, F4C, which expresses *fur* gene controlled by isopropyl-beta-D-thiogalactopyranoside (IPTG)-inducible *lac* promoter, partially complemented the WT phenotype.

The hypersensitivity of F4 to H_2_O_2_ may be due to increased intracellular free iron concentration resulting from de-regulation of iron transport [11], or to decreased enzyme activity as part of an “oxidative stress response”. To assess these possibilities, we tested viability of the three strains in the presence of 1 µg ml^−1^ SNG. SNG is a quinine-related antibiotic that is cyclically reduced and oxidized inside bacteria, leading to production of superoxide and hydroxyl radicals which cause DNA damage and eventual cell death [12], [13]. It is frequently used to assess free iron levels in bacteria [14]–[16]. Higher concentrations of free intracellular iron enhance the effect of SNG and the degree of damage to cells [17].

**Figure 3:**
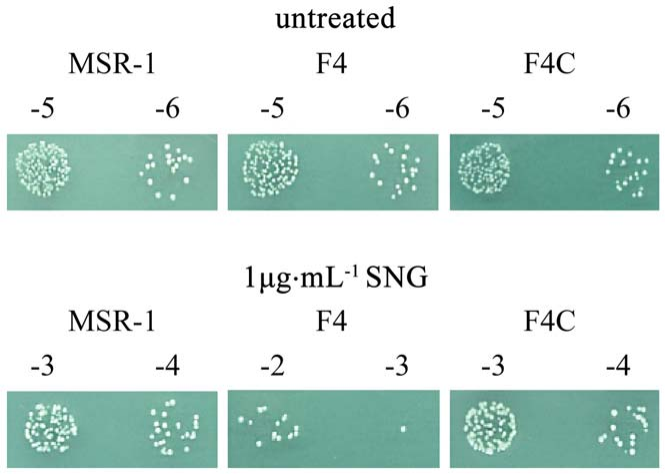
Comparative sensitivity of WT, F4, and F4C strains to streptonigrin (SNG). Cells were treated without or with 1 µg/ml SNG for 5 days at 30°C. Cultures were diluted and spotted on agar plates with SLM. Numbers above each image indicate 10-fold serial dilutions.

Non-treated WT, F4 and F4C without treating showed very similar numbers colonies on plates. Following SNG treatment, growth of F4 cells was greatly reduced, while that of WT and F4C was not (**Figure 3**). The concentration of intracellular free iron (Fe^2+^) in F4 is therefore higher than that in WT. We presume that loss of Fur disrupts homeostasis of ferrous iron in cells, also this explains the high H_2_O_2_ sensitivity of F4.

### F4 strain has reduced cellular iron level and ability to synthesize magnetosomes

**Figure 4:**
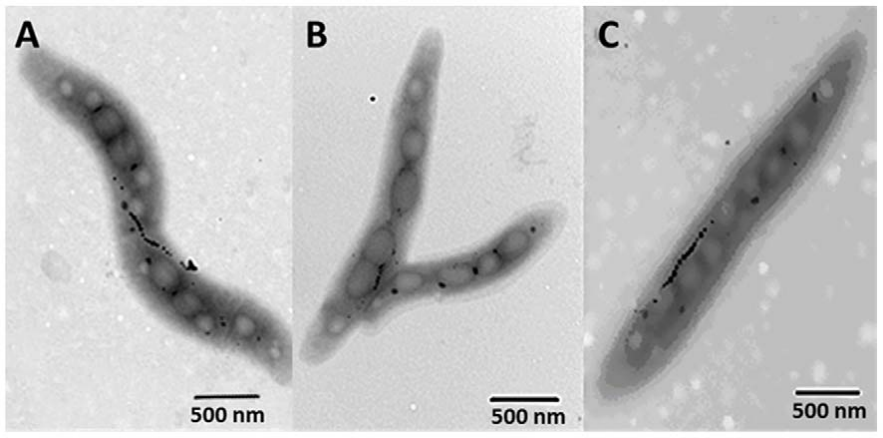
TEM micrographs of WT (A), F4 (B), and F4C (C) strains. Cells were grown in SLM added with 60 µM ferric citrate for 36 h. Bar, 500 nm.

The process of magnetosome formation in *M. gryphiswaldense* is closely related to iron uptake [18]. To assess the effect of *fur* mutation on magnetosome formation, we measured total cellular iron content and magnetosome yield of WT, F4 and F4C cells following 24 h culture. The three strains accumulated 3.8±0.9, 2.3±0.4, and 3.2±0.5 mg magnetosomes per gram cell dry weight, and contained 0.58±0.11%, 0.37±0.01% and 0.46±0.01% iron (as dry weight) respectively. It is clear that F4 synthesis 40% less than wild type. The reduced magnetosome formation of F4 was confirmed by TEM micrography (**Figure 4**).

### Fur is the iron-responsive regulator of four genes involved in iron or oxygen metabolism in *M. gryphiswaldense*

#### Fur regulates transcription levels of *feoAB1*, *feoAB2*, *katG*, and *sodB*

F4 and WT strains differ in their sensitivity to H_2_O_2_ and SNG, factors which also affect iron and oxygen metabolism. We therefore examined the regulatory effect of Fur on four key genes involved in iron or oxygen metabolism: *feoAB1* (EF120624.1), which is necessary for magnetosome formation [19]; *feoAB2* (MGR_1447, 1446), which is probably related to other metal ion uptake protein (data not shown); *katG* (MGR_4274), which encodes catalase-peroxidase; *sodB* (MGR_3446) which encodes superoxide dismutase. The latter two are typical “oxidative stress response” genes.

WT and F4 cells were cultured under high-iron and low-iron conditions, and transcription levels of the above four genes were tested. Under high-iron condition, mRNA levels of *katG* and *sodB* were higher in F4 than in WT (**Table 1** **,** ***fur***), suggesting that the enzyme activities were increased in mutants and mRNA level of *feoAB1* was 7.66-fold higher in F4 than in WT. Results for *feoAB2* were similar, although this gene is probably not directly involved in ferrous iron transport. These findings suggest again that the H_2_O_2_ sensitivity of F4 is due to higher level of intracellular free iron, but on a transcriptional basis.

**Table 1:** Iron-responsive Fur regulon in *M. gryphiswaldense*.

Gene	Function	RT-PCR		Product
		DP	*fur*	
EF120624.1	Iron acquisition system: Fe^2+^ transporter (Feo)	1.08	7.66	Ferrous iron transport protein(FeoAB1)
MGR_1447-1446	Iron acquisition system: Fe^2+^ transporter (Feo)	−1.32	4.55	Ferrous iron transport protein(FeoAB2)
MGR_3446	Catalyze dismutation of superoxide anions	−1.48	2.09	Superoxide dismutase
MGR_4274	Catalase-peroxidase	1.94	1.76	Catalase-peroxidase

DP, the comparison of mRNA from WT cells treated with 30 µM iron chelator 2, 2′-dipyridyl (DIPy) and cells added with 60 µM ferric citrate.

*fur*, the comparison of mRNA expression in the *fur* mutant (F4) and WT grown under high-iron condition.

Positive and negative numbers indicate fold increase or decrease, respectively.

mRNA levels of the four genes were not very different in WT under high-iron vs. low-iron conditions (**Table 1**, DP). The normal balance among these genes in cells is disrupted by loss of *fur*. Thus, *fur* regulates these four genes *in vivo*, and maintains the balance among them during environmental changes.

#### Fur directly combines with the promoters of *feoAB1*, *feoAB2*, *katG*, and *sodB* in vivo

Real-time RT-PCR results showed that expression of these four genes is repressed in MSR-1. Fur is typically a global regulator and may affect gene expression in a direct or indirect manner. We performed ChIP assay to investigate how Fur_MSR_ regulates these four genes. ChIP assay determines whether a specific protein interacts with a particular piece of chromatin in vivo. The complexes of DNA fragments and protein are immunoprecipitated by the corresponding antibody [20].

**Figure 5:**
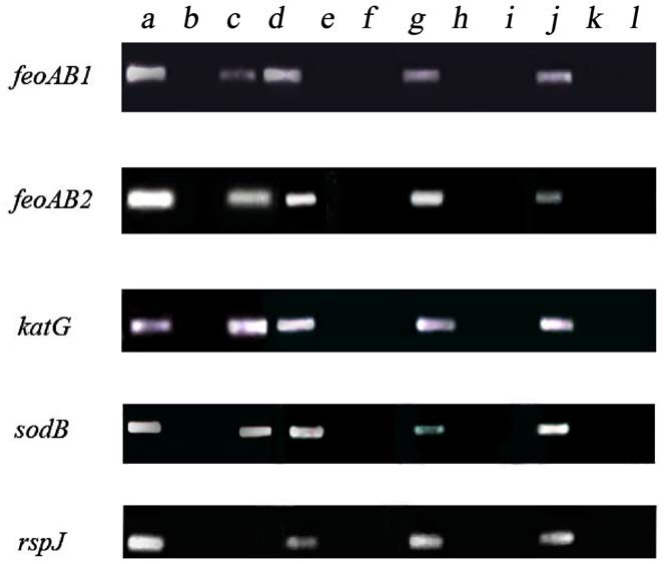
Detection of ChIP DNAs from WT and F4 strains under low-iron and high-iron conditions. **a, d, g, j**: positive controls (input). **b, e, h, k**: negative controls (no antibody). **c**: high-iron WT. **f**: low-iron WT. **i**: high-iron F4. **l**: low-iron F4. Primer of *rpsJ* gene (Table 3) is included as an additional negative control which codes for a conserved 30S ribosomal S10 protein and is not regulated by Fur [32].

We performed ChIP assay with polyclonal anti-Fur_MSR_ antibodies and oligos to amplify the promoter sequences of *feoAB1*, *feoAB2*, *katG* and *sodB* in WT and F4 cultured under low-iron and high-iron conditions. Results showed that the promoter regions were amplified only by DNAs immunoprecipitated from WT cultures under high-iron condition (**Figure 5**, lane c) and no promoter fragments were amplified from ChIP DNAs of low-iron WT, or high-iron or low-iron F4 (**Figure 5**, lanes f, i, l). We conclude that Fur directly interacts with and down-regulates *feoAB1*, *feoAB2*, *katG*, and *sodB* through their promoters.

## Discussion

Fur protein directly or indirectly regulates intracellular iron storage and utilization, as well as iron uptake, in many types of bacteria. For magnetotactic bacteria, iron is essential for synthesis of magnetite (Fe_3_O_4_) crystals, *i.e.*, magnetosomes. Although there have been several studies of iron uptake systems in magnetotactic bacteria [21], [22], it remains unclear whether Fur is involved in biomineralization of magnetosomes, and which particular genes are regulated by Fur. We therefore used genetic complementation to confirm the presence of a Fur homologue in *M. gryphiswaldense* and functionally characterized the protein.

We showed previously that *M. gryphiswaldense* has a gene closely homologous to *fur* (GenBank accession # ABE73150), and that mutation of this gene results in decreased magnetosome formation and increased H_2_O_2_ sensitivity, a common trait of bacterial perR mutants [23]. These findings, together with SWISS-MODEL analysis of protein structure (http://swissmodel.expasy.org/) (data not shown), suggested that the protein product of this *fur*-like gene functions as a repressor of peroxide stress response (PerR), rather than an iron-responsive gene regulator.

Our subsequent study showed that the *M. gryphiswaldense* genome contains four *fur*-homologue genes: MGR_1305, MGR_1314, MGR_1399 (corresponding to ABE73150), and MGR_3480. The protein encoded by MGR_1314 was identified as a functional Fur homologue, since it functionally complemented the *fur* mutant of *E. coli* H1780.

To determine whether Fur_MSR_ functions as an iron-responsive transcriptional repressor *in vivo*, we constructed a *fur* mutant of *M. gryphiswaldense* strain MSR-1, termed F4 and its complementary F4C. F4 was highly sensitive to H_2_O_2_ and to SNG, suggesting that the mutation reduces activity of the enzymes catalase and superoxide dismutase, or increases concentration of intracellular free iron. The MSR-1 genome contains two *feo* operons: *feoAB1*, which is involved in ferrous iron uptake [19], and *feoAB2* (MGR_1447-1446) which is annotated as a *feo* operon by National Center for Biotechnology Information(NCBI)web site. Quantitative real-time RT-PCR analysis indicated that these effects of *fur* mutation were due not to altered activity of catalase or SOD, but rather to increased intracellular free iron concentration, resulting from up-regulation of *feoAB* under high-iron condition. The qRT-PCR also indicated that *feoAB1*, *feoAB2*, *katG* and *sodB* genes are all regulated by Fur, although the situation for *katG* remains unclear. The ratio of *katG* between low-iron vs. high-iron WT is nearly 2. Further ChIP assay indicated that all four genes are regulated by Fur *in vivo*.

It is reported that “*feoAB1* express lower in *fur* mutant than WT under both iron-rich and responsive conditions” [24]. In our research the ChIP analysis showed that Fur_MSR_ binds to the promoters of the two *feo* operons and also to those of *kat*G and *sodB*, indicating that it can regulate all four genes. Analysis of the four promoters revealed a conserved 19 bp motif with palindromic symmetry, and a shared consensus 5′-3′ sequence (data not shown).

In *E.coli*, it is proved *sodB* is positive regulated by Fur and by indirect situation [25]. Interestingly in MSR-1, *sodB* is directly negative regulated by Fur, which means that in fur mutant it can resist more Fenton reaction. This may explain why MSR-1 can survive in a high free iron condition.

Total magnetosome formation was significantly reduced in the *fur* mutant. It is reported that the process of magnetosome formation in *M. gryphiswaldense* is closely related to iron uptake [18]. But in our research, the mutant (F4) has lower resistance to SNG. So the intracellular free iron (Fe^2+^) of mutant is higher than the wild type and the complementary (F4C). As this point we speculate that some key genes of magnetosome formation especially the genes corresponding to iron transport are blocked by the disruption of Fur. Though it is reported that their *M. gryphiswaldense fur* mutant showed only one MAIs protein (magnetosome islands) Mms6 has difference in expression level. This protein is reported to affect magnetosome crystal formation in vitro [24]. According to our research it is apparently insufficient. It is interested to further research whether other MAI genes and other magnetosome formation genes outside of MAI regulated by Fur. Maybe the expression differences only show in a certain growth period. Results of the present study clearly indicate that Fur protein functions as an important regulator of iron and oxygen metabolism in *M. gryphiswaldense* strain MSR-1, and also affects magnetosome formation. Studies to clarify the connection between these roles are in progress.

## Materials and Methods

### Bacterial strains and growth conditions

Bacterial strains and plasmids used in this study are summarized in **Table 2**. *E. coli* strains were cultured in Luria-Bertani (LB) medium at 37°C. For complementation of *E. coli* H1780, we used LB medium supplemented with 100 µM FeCl_3_ for high-iron condition, or with the iron chelator 2, 2′-dipyridyl (DIPy; Sigma), 200 µM, for low-iron condition. When required, antibiotics were added at the following concentrations (µg·ml^−1^): ampicillin (Amp) 100; tetracycline (Tc) 12.5; kanamycin (Km) 50; chloramphenicol (Cm) 25; gentamycin (Gm) 20.

**Table 2:** Bacterial strains and plasmids used in this study.

Strain or plasmid	Description	Source or reference
Strain		
*E.coli*		
DH5α	*endA1 hsdR17 [r-m+] supE44 thi-1 recA1 gyrA [NalR] relA1* Δ *[lacZYA-argF] U169 deoR [Ø80*Δ*{lacZ} M15]*	Sambrook *et al.*, 2001
H1780	*araD139*Δ^a^ *argF-lacU169rpsL150 relA1 flbB5301deoC1 ptsF25 rbsR fiu::lacZ* fusion lacking *fur*, Sm^r^, Km^r^	Hantke *et al.*,1987
K12		
BL21(DE3)	*hsdS gal* (λcI*ts857 ind-1 S*am7 *nin-5 lacUV5-T7 gene 1*)	Studier, F.W. *et al.*, 1986
S17-1	*thi endA recA hsdR* with RP4-2-Tc::Mu-Km::Tn*7* integrated in chromosome, Sm^r^	Simon *et al.*, 1983
*M. gryphiswaldense*		
MSR-1 (DSM 6361)	Wild-type	Schleifer D *et al*., 1991
F4	mutant type (*fur::Gm*)	Present study
F4C	F4 with pRKFC	Present study
Plasmids		
pUC18	Cloning vector, Ap^r^	Messing
pET-28a-c(+)	Expression vector T7 promoter, Km^r^	Novagen
pSUP202		Simon *et al.*, 1983
pRK415	Broad host range cloning vector, Tc^r^	Scott *et al.*, 2003
pEF	0.6 kb *Bam*HI-*Hind*III fragment (encoding *E. coli* K12 Fur) with *fur* promoter cloned in BamHI-HindIII sites of pUC18, Ap^r^	Present study
pRF	0.6 kb *Bam*HI-*Hind*III fragment (encoding *M. gryphiswaldense* MSR-1 Fur) cloned in *Bam*HI-*Hind*III sites of pUC18, Ap^r^	Present study
pETRF	pET-28a-c(+) derivative, expresses the His-tag protein of *M. gryphiswaldense* Fur. Km^r^	Present study
pUDG	pSUS202 containing 3.2 kb fragment with *fur*::Gm	Present study
pRKFC	pRK415 containing 0.6 kb *fur* gene from *M. gryphiswaldense* MSR-1	Present study

*M. gryphiswaldense* strains were cultured in sodium lactate medium (SLM) at 30°C, as described previously [19]. 100 ml liquid culture was placed in a 250-ml serum bottle plugged with rubber stopper, and incubated in a rotary shaker at 100 rpm. For plate culture, diluted liquid culture was spread on solid agar medium, and plates were sealed with Parafilm to produce microaerobic condition and incubated at 30°C [26]. For high-iron condition medium was supplemented with ferric citrate (final concentration 60 µM), and for low-iron condition medium was supplemented with DIPy (30 µM). When required, antibiotics were added at 5 µg·ml^−1^: nalidixic acid (Nx); Tc; Cm; Gm.

### Null strain construction and complementation

MSR-1 *fur* mutant was constructed by allelic exchange (**Figure S3**). Sequences ∼1.2 kb upstream and downstream of *fur* were amplified using primer sets rfuup/rfulow, and rfdup/rfdlow (**Table S1**). Amplified DNA fragments were cut with appropriate restriction enzymes, and ligated into the suicide vector pSUP202 to form pFUD. The gentamycin resistance cassette from pUCGm was inserted as a *Kpn*I fragment into the *Kpn*I site of pFUD, and a plasmid containing the gentamycin cassette oriented in the same direction as that of *fur* gene transcription was selected, yielding pUDG. pUDG was conjugated into MSR-1 wild-type, using *E. coli* S17-1 as donor strain.

Bi-parental conjugation of *M. gryphiswaldense* with *E. coli* S17-1 was performed in SLM, as described previously [19]. Transformants were replica plated on medium containing either Cm or Gm. Knockouts that grew only in the presence of Gm were selected. Disruption of *fur* gene was confirmed by PCR analysis. The mutant was termed F4.

To construct a plasmid complementary with *fur* that can be transcribed from a *lac* promoter, full-length WT *fur* gene and its ribosomal binding sequence were amplified from MSR-1 genomic DNA with primers fcup and fclow (**Table S1**), using *Pfu* DNA polymerase, and cloned into *Hin*dIII-*Eco*RI sites of expression vector pRK415, creating recombinant plasmid pRKFC. The cloned DNA region was confirmed by automated DNA sequencing. The pRK415 was introduced into F4. The complemented strain of F4 was termed F4C.

### Complementation *E. coli fur* mutant

MGR_1314 and its ribosomal binding sequence were PCR-amplified from chromosome with primers rfup and rflow (**Table S2**). The single PCR product was digested at primer-derived restriction sites (*Bam*HI, *Hin*dIII), and then cloned into high-copy-number vector pUC18, giving rise to pRF. Similarly, complete *E. coli fur* gene amplified with primers efup and eflow (**Table S2**) was cloned into pUC18 to create pEF. Plasmid pRF, as well as pEF vector (positive control) and pUC18 vector (negative control), were transformed into H1780. β-Galactosidase activity was determined as described by Miller [27], with cells grown under high or low-iron condition. Triplicate assay was performed for each sample.

### Purification of recombinant Fur, and preparation of anti-Fur antibodies

MSR-1 *fur* gene was amplified by PCR using *Pfu* polymerase with primers hrfup and hrflow (**Table S2**), cloned into pET-28a^+^ at *Nde*I and *Hin*dIII sites, and confirmed by automated sequencing. The plasmid was transformed into *E. coli* strain BL21 (DE3) for protein expression, and cells were grown in 100 ml LB medium supplemented with 50 µg·mL^−1^ Km, at 37°C. When the culture reached OD_600_ 0.4–0.6, 1 mM IPTG was added to induce Fur protein expression. Cells were grown 4 h, harvested by 13,000*g centrifugation at 4°C, and the pellet was suspended in 10 mL lysis buffer [50 mmol/L NaH_2_PO_4_, pH 8.0, 300 mmol/L NaCl, 10 mmol/L imidazole, 1 mmol/L phenylmethanesulfonyl fluoride (PMSF)]. The cell suspension was lysed by sonication, and centrifuged at 13,000×g for 20 min at 4°C. The combination protein containing a 6-histidine tag (His-Tag) was purified by affinity chromatography on nickel (Ni) column (Qiagen), and the supernatant was applied to Ni-NTA agarose equilibrated with lysis buffer. The column was washed with 10 column volumes washing buffer (50 mmol/L NaH_2_PO_4_, pH 8.0, 300 mmol/L NaCl, 20 mmol/L imidazole), the His-Tag-N-terminal protein was eluted with elution buffer containing 250 mmol/L imidazole. Eluted fractions were analyzed by 12% SDS-PAGE (**Figure S4**). The purified protein was dialyzed against buffer (25 mmol/L Tris-HCl, pH 8.0, 50 mmol/L NaCl, 10 mmol/L MgCl_2_, 0.1 mmol/L dithiothreitol, 5% (v/v) glycerol) and stored in this buffer at −20°C.

Polyclonal anti-Fur antibodies were prepared by injection of purified Fur protein into rabbits, at Beijing Protein Institute, China.

### Strains senditivity to H_2_O_2_ and SNG

MSR-1 strains were grown in SLM until stationary phase. Cultures were adjusted to the same OD_565_, and diluted 1∶10 in 100 ml SLM containing 1 mM H_2_O_2_. Cells were grown with shaking at 30°C for 24 h, with frequent measurement of OD_565_.

SNG sensitivity assay was performed as described previously [15], with slight modification. SNG was prepared as a 1 mg/ml stock solution in dimethyl sulfoxide. Each strain was cultured in SLM at 30°C until stationary phase. Cultures added with SNG (1 µg/ml), or with equivalent concentration of dimethyl sulfoxide as control, were incubated in a rotary shaker (100 rpm, 2 h, 30°C), and serially diluted 10-fold. 10 µl of each dilution was spotted on agar plate with SLM, and incubated 7 days at 30°C. Each strain was tested in triplicate, and the experiment was repeated twice.

### Iron content and magnetosome yield

WT, *fur* mutant strain (F4) and complementation strain (F4C) of MSR-1 were grown in SLM supplemented with 60 µM ferric citrate. Total cellular iron content was measured by atom absorption spectrophotometry [28]. Magnetosome yield was determined as described by Sun *et al.* [29]. Measurements were taken from triplicate cultures.

### Transmission electron microscopy

WT, F4, and F4C strains were grown in SLM added with 60 µM ferric citrate for 36 h. Cells were fixed with 2.5% glutaraldehyde. Cell suspensions were coated on copper grids and observed directly by transmission electron microscopy (Model H-8000, Hitachi, Japan).

### Quantitative real-time RT-PCR (qRT-PCR)

WT and F4 strains were grown in SLM to OD_565_ 0.5, and culture was split. One half was added with 30 µM DIPy (low-iron condition); the other half was added with 60 µM ferric citrate (high-iron condition). Growth was continued 2 h at 30°C, and cells were harvested. Total cellular RNA was isolated using Trizol reagent (Invitrogen), and digested with RNase-free DNase I (Promega) for 30 min at 37°C. RNA quality and quantity were evaluated by spectrophotometric readings at wavelength 260 and 280 nm. Successful DNase treatment was confirmed by PCR using r-Taq DNA polymerase (Takara), and 16sup and 16slow primers (**Table 3**), and RNA extracted from each sample was reverse-transcribed into cDNA using M-MLV reverse transcriptase (Invitrogen). Reaction took place in a final volume of 20 µl containing 4 µl first strand buffer (5×), 1 µl dNTPs mix (2.5 mM/µl), 2 µl oligo (dT), 15 µl primer (500 µg/ml; Promega), 0.5 µl RNase inhibitor (40 U/µl; Promega), 2 µl DL-Dithiothreitol (DTT, 0.1 M; Invitrogen), 1 µl M-MLV reverse transcriptase (200 U/µl; Invitrogen), 2.5 µg template RNA, and RNase-free water.

**Table 3:** Primers of qRT-PCR.

Gene	Function of gene	Sequence of primer (5′-3′)	PCR product size
*feoAB1* (EF120624.1)	Fe^2+^ transport system protein	feo1up:TGGTCCACGAGCATGATGAG feo2low:ATGGCACCCAGGCTGAAAGT	226 bp
*feoAB2* (MGR_1447-1446)	Fe^2+^ transport system protein	feo2up: GAGGAACCCGACATCATCA feo2low:TCAGGGCCAGCGATATCTT	100 bp
*katG* (MGR_4274)	Catalase- peroxidase	cpup: TGAACGACGAGGAAACGGT cplow: CCACCAGTCATAGCCCAACAG	257 bp
*sodB* (MGR_3446)	Superoxide dismutase	sdup: CGCCTATGTGACCAACCTGAA sdlow: AATTCCTCGGCGAACTTTTC	252 bp
16S ribosomal RNA	16S ribosomal protein	16sup: CTTGTGATAACGCCAAACCC 16slow: TTGCCGCTACCGATACTCTT	239 bp

qRT-PCR was performed in a Roche Lightcycler 1.2 RT-PCR System (Roche), using Lightcycler-Faststart DNA master SYBR green I PCR kit (Roche) according to manufacturer's instructions. Primers used are listed in **Table 3**. Specific primers were designed to yield ∼100–300 bp sequences. qRT-PCR mixture (total volume 20 µl) contained 14.2 µl water, 1.6 µl MgCl_2_, 0.6 µl of each primer (10 µM), 2 µl Fast Start DNA Master SYBR Green I, and 1 µl RT product. Steps of PCR were: denaturation (95°C, 10 min), 40 amplification cycles (each 95°C for 15 sec), melting temperature for each primer pair (15 sec), extension (72°C, 20 sec), and plate reading for fluorescence data (76°C). To evaluate specificity of the amplified product, melting curves were analyzed from 75 to 95°C, followed by 1.5% agarose gel electrophoresis. Absence of genomic DNA contamination was confirmed by absence of reverse-transcribed total RNA samples from the processing reaction.

Fold amplification was calculated by comparative threshold cycle (CT) method [30], [31]. To correct for sampling errors, expression level of each gene was normalized by dividing by expression level of 16S rRNA transcript. Data from three replicates were averaged.

### Chromatin immunoprecipitation (ChIP) assay

ChIP Assay Kit (Upstate Biotechnology, cat # 17-295, lot # 29633) was used, per manufacturer's instructions, with some modification. WT and F4 strains were grown in SLM to OD_565_ 0.9, culture was split, and two halves were treated with DIPy or ferric citrate to elicit low-iron or high-iron condition, as described in the preceding section. Culture was continued in 1 L SLM until log phase. Sonication conditions for chromatin: Set sonicator (JY92-II, Ningho Scentz Biotechnology Co. Ltd, China) at 150W. 5 mL nuclear lysis samples pulses 240 of 3 sec (10 sec intervals). Average chromatin fragment size: 200–1000 bp (**Figure S5**). The amount of rabbit polyclonal anti-Fur antibodies (produced as described above) added to cross-linked chromatin was determined empirically. No antibody negative control samples were included. ChIP DNAs were used as templates for PCR amplification, to determine whether the DNA site in question was cross-linked to Fur. Sequences of PCR primers used to analyze genes are listed in **Table 4**.

**Table 4:** Primers for ChIP PCR detection.

Gene	Function of gene	Sequence of primer (5′-3′)	PCR product size
*feoAB1* (EF120624.1)	Fe^2+^ transport system protein	pforward: CGGGGTACCACATAGAATTCATGCTGC promoter1: AAACTGCAGATCTTGACCTGCTGATCCA	553 bp
*feoAB2* (MGR_1447-46)	Fe^2+^ transport system protein	p2f: CATGCCGGCGAAACCAAGCGC p2l: GGCGGCGCCTCCGATGGG	205 bp
*katG* (MGR_4274)	Catalase-peroxidase	katGpf: CGCACGATCGTCATTTCCTC katGpl: GTCGCTCTCCCATTCAACCAAT	282 bp
*sodB* (MGR_3446)	Superoxide dismutase	sodBpf: GCCCGCAAGATAATTTCGATACAG sodBpl: CGGGATATGCATAATGTTGAAGGG	529 bp
*rpsJ* (MGR_3815)	30S ribosomal protein S10	rspJpf: GCCGATCATCGAGTAGTCCT rspJpl: CGTTAAATCGGATCGGCGC	249 bp

## Supporting Information

Figure S1 Sequence alignment generated by the ClustalW program between MGR_1314, accession # CAM76422 and five related Fur sequences (B.a, *Brucella abortus*, accession # AAB81452; E.c, *E. coli* O157:S7, accession # NP_286398; K.p, *Klebsiella pneumoniae*, accession # AAB51077; P.a, *Pseudomonas aeruginosa*, accession # AAC05679; A.f, *Acidithiobacillus ferrooxidans*, accession # AAR85472).
(DOC)

Figure S2 Tertiary structure of Fur. **A:** From *M. gryphiswaldense* MSR-1. **B:** From *Pseudomonas aeruginosa*.
(DOC)

Figure S3 Procedure (schematic) for construction of *fur* mutant.
(DOC)

Figure S4 Overexpression and purification of His-tag Fur. Crude extracts and purified recombinant proteins were analyzed on 12% SDS-polyacrylamide gel. **A:** Lane 1, non-induced *E. coli* cells carrying pET-Fur. Lane 2, IPTG-induced *E. coli* with pET-Fur. M, standard protein markers (97.4, 66.2, 43, 31, 20.1, 14 kDa). **B:** Lane 1, purified recombinant His-tag Fur protein eluted from Ni-NTA column. M, standard protein markers the same as in A.
(DOC)

Figure S5 Optimization of DNA shearing. Sonication conditions for chromatin as described under Materials & Methods “Chromatin immunoprecipitation (ChIP) assay”.
(DOC)

Table S1 Primers for construction and complementation of *fur* mutant.
(DOC)

Table S2 Primers to replicate *fur* gene.
(DOC)
