# Supplementary material for: Fur in Magnetospirillum gryphiswaldense Influences Magnetosomes Formation and Directly Regulates the Genes Involved in Iron and Oxygen Metabolism
Source: PLoS One. 2012 Jan 4;7(1):e29572. doi: 10.1371/journal.pone.0029572 (PMC3251581; doi:10.1371/journal.pone.0029572)
Supplement: Figure S1 — Sequence alignment generated by the ClustalW program between MGR_1314, accession # CAM76422 and five related Fur sequences (B.a, Brucella abortus, accession # AAB81452; E.c, E. coli O157:S7, accession # NP_286398; K.p, Klebsiella pneumoniae, accession # AAB51077; P.a, Pseudomonas aeruginosa, accession # AAC05679; A.f, Acidithiobacillus ferrooxidans, accession # AAR85472). (DOC) [file pone.0029572.s001.doc]

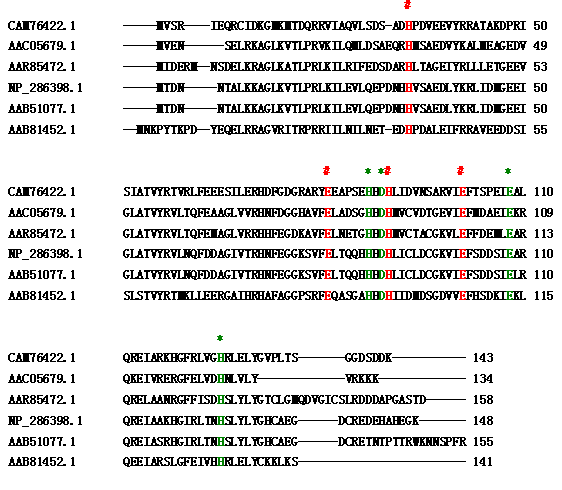


**Supporting Figure S 1 (Lei Qi, *et al*.)**

**Supporting Figure S1.** Sequence alignment generated by the ClustalW program between MGR_1314, accession # CAM76422 and five related Fur sequences (B.a, *Brucella* *abortus*, accession # AAB81452; E.c, *E. coli* O157:S7, accession # NP_286398; K.p, *Klebsiella pneumoniae*, accession # AAB51077; P.a, *Pseudomonas aeruginosa*, accession # AAC05679; A.f, *Acidithiobacillus ferrooxidans*, accession # AAR85472).
