# Supplementary material for: Fur in Magnetospirillum gryphiswaldense Influences Magnetosomes Formation and Directly Regulates the Genes Involved in Iron and Oxygen Metabolism
Source: PLoS One. 2012 Jan 4;7(1):e29572. doi: 10.1371/journal.pone.0029572 (PMC3251581; doi:10.1371/journal.pone.0029572)
Supplement: Figure S2 — Tertiary structure of Fur. A: From M. gryphiswaldense MSR-1. B: From Pseudomonas aeruginosa. (DOC) [file pone.0029572.s002.doc]

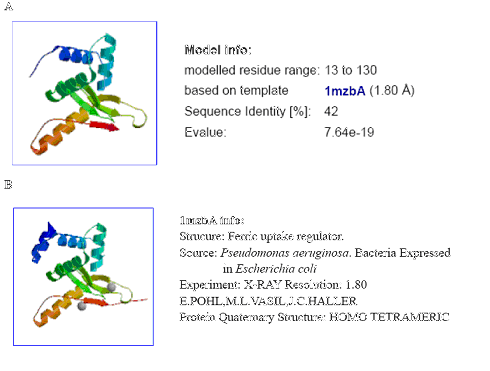


Structure: predicted Ferric uptake regulator of *Magnetospirillum gryphiswaldense* strain MSR-1

Software: Swiss-model ([http://www.expasy.org](http://www.expasy.org/))

GenBank: CU459003.1 (MGR_1314)

Structure: Ferric uptake regulator

Source: *Pseudomonas aeruginosa.* Bacteria Expressed in *Escherichia coli*

NCBI Reference Sequence: NP_253452.1

**Supporting Figure S2**  **(Lei Qi, *et al*.)**

**Supporting Figure S2**. Tertiary structure of Fur. **A:** From *M. gryphiswaldense* MSR-1. **B:** From *Pseudomonas aeruginosa*.
