# Supplementary material for: Fur in Magnetospirillum gryphiswaldense Influences Magnetosomes Formation and Directly Regulates the Genes Involved in Iron and Oxygen Metabolism
Source: PLoS One. 2012 Jan 4;7(1):e29572. doi: 10.1371/journal.pone.0029572 (PMC3251581; doi:10.1371/journal.pone.0029572)
Supplement: Figure S3 — Procedure (schematic) for construction of fur mutant. (DOC) [file pone.0029572.s003.doc]

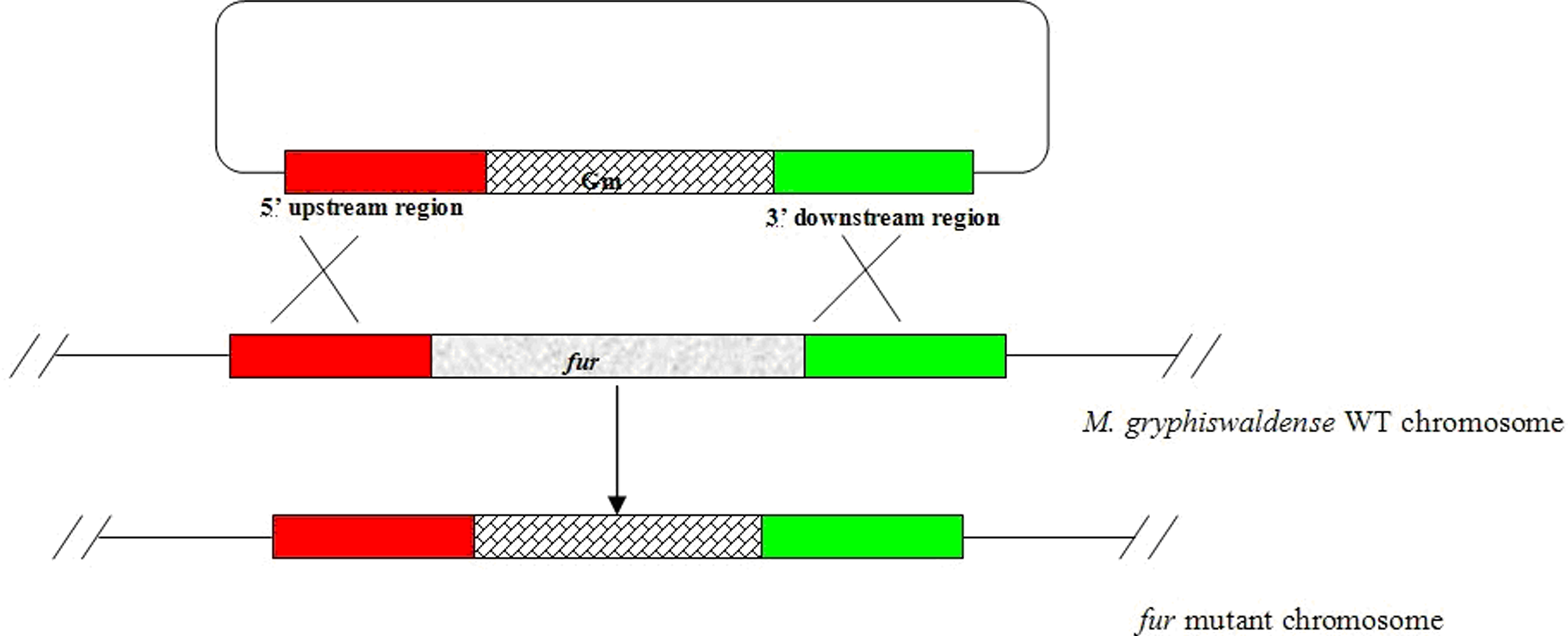


**Supporting Figure S3 (Lei Qi, *et al*.)**

**Supporting Figure S3.**  Procedure (schematic) for construction of *fur* mutant.
