# Supplementary material for: Fur in Magnetospirillum gryphiswaldense Influences Magnetosomes Formation and Directly Regulates the Genes Involved in Iron and Oxygen Metabolism
Source: PLoS One. 2012 Jan 4;7(1):e29572. doi: 10.1371/journal.pone.0029572 (PMC3251581; doi:10.1371/journal.pone.0029572)
Supplement: Figure S4 — Overexpression and purification of His-tag Fur. Crude extracts and purified recombinant proteins were analyzed on 12% SDS-polyacrylamide gel. A: Lane 1, non-induced E. coli cells carrying pET-Fur. Lane 2, IPTG-induced E. coli with pET-Fur. M, standard protein markers (97.4, 66.2, 43, 31, 20.1, 14 kDa). B: Lane 1, purified recombinant His-tag Fur protein eluted from Ni-NTA column. M, standard protein markers the same as in A. (DOC) [file pone.0029572.s004.doc]

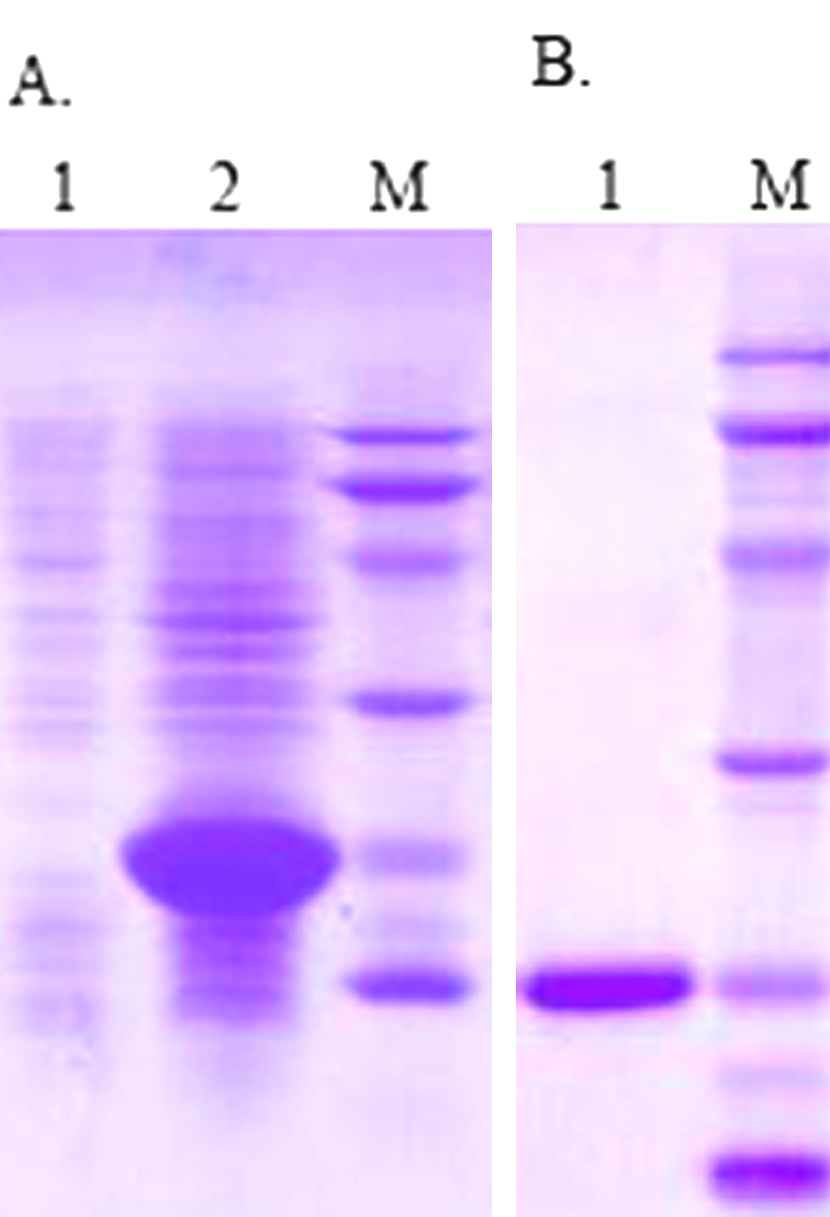


**Supporting Figure S4 (Lei Qi *et al*.)**

**Supporting Figure S4** Overexpression and purification of His-tag Fur. Crude extracts and purified recombinant proteins were analyzed on 12% SDS-polyacrylamide gel. **A:** Lane 1, non-induced cells of *E. coli* carrying pET-Fur. Lane 2, IPTG-induced *E. coli* cells with pET-Fur. M, standard protein markers (97.4, 66.2, 43, 31, 20.1, 14 kDa). **B:** Lane 1, purified recombinant His-tag Fur protein eluted from Ni-NTA column. M, standard protein markers the same as in A.
