# Supplementary material for: Fur in Magnetospirillum gryphiswaldense Influences Magnetosomes Formation and Directly Regulates the Genes Involved in Iron and Oxygen Metabolism
Source: PLoS One. 2012 Jan 4;7(1):e29572. doi: 10.1371/journal.pone.0029572 (PMC3251581; doi:10.1371/journal.pone.0029572)
Supplement: Figure S5 — Optimization of DNA shearing. Sonication conditions for chromatin as described under Materials & Methods “Chromatin immunoprecipitation (ChIP) assay”. (DOC) [file pone.0029572.s005.doc]

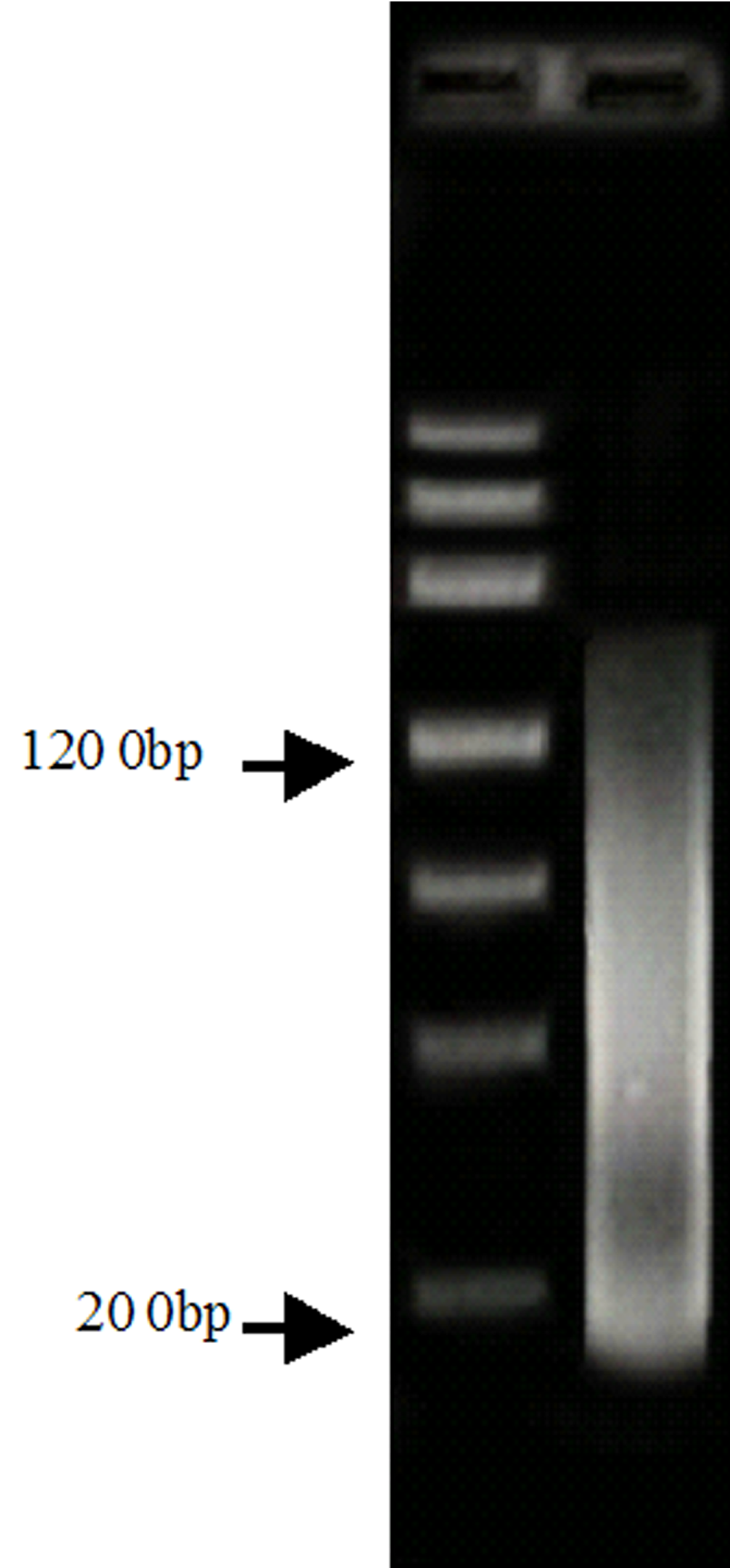


**Supporting Figure S5 (Lei Qi, *et al*.)**

**Supporting Figure S5.** Optimization of DNA shearing. Sonication conditions for chromatin as described under Materials & Methods/ "Chromatin immunoprecipitation (ChIP) assay".
