# Supplementary material for: Fur in Magnetospirillum gryphiswaldense Influences Magnetosomes Formation and Directly Regulates the Genes Involved in Iron and Oxygen Metabolism
Source: PLoS One. 2012 Jan 4;7(1):e29572. doi: 10.1371/journal.pone.0029572 (PMC3251581; doi:10.1371/journal.pone.0029572)
Supplement: Table S1 — Primers for construction and complementation of fur mutant. (DOC) [file pone.0029572.s006.doc]

**Table S1. Primers for construction and complementation of *fur* mutant**

| Number | Primer name | Sequence (5’→ 3’) | Enzyme site |
| --- | --- | --- | --- |
| 1 | rfuup | GAGGATCCTTTGCGACAAATCCGGGCTGTTG | *Bam*HI |
| 2 | rfulow | GAGGTACCGCCCTTCGTCAGGATTATCGGAC | *Kpn*I |
| 3 | rfdup | GAGGTACCGCCATTTAATCAGGCGAGGATAC | *Kpn*I |
| 4 | rfdlow | GAAAGCTTAGCGAATCACCGCCCTTCAGG | *Hin*dIII |
| 5 | fcup | GAAAGCTTCCTTTGGTTTATGATCCCCG | *Hin*dIII |
| 6 | fclow | GAGAATTCGTTCCTTGGCTATTTGTCGTC | *Eco*RI |
