# Supplementary material for: Fur in Magnetospirillum gryphiswaldense Influences Magnetosomes Formation and Directly Regulates the Genes Involved in Iron and Oxygen Metabolism
Source: PLoS One. 2012 Jan 4;7(1):e29572. doi: 10.1371/journal.pone.0029572 (PMC3251581; doi:10.1371/journal.pone.0029572)
Supplement: Table S2 — Primers to replicate fur gene. (DOC) [file pone.0029572.s007.doc]

Table **S2**. Primers to replicate *fur* gene

| Number | Primer name | Sequence (5’→ 3’) |
| --- | --- | --- |
| 1 | rfup | GAGGTACCTCTTTTGCCGCGTCTGATCTC |
| 2 | rflow | GAAAGCTTCGTTCCTTGGCTATTTGTCGTC |
| 3 | efup | GAGGATCCTTCAAGTGGCCTTGCCGTTG |
| 4 | eflow | GAAAGCTTTTGGCTTTTCTCGTTCAGGC |
| 5 | hrfup | GACATATGGTTTCGCGTATTGAAC |
| 6 | hrflow | GAAAGCTTCGTTCCTTGGCTATTTGTCGTC |
